# Bone edema on magnetic resonance imaging is highly associated with low bone mineral density in patients with ankylosing spondylitis

**DOI:** 10.1371/journal.pone.0189569

**Published:** 2017-12-14

**Authors:** Danmin Wang, Zhiduo Hou, Yao Gong, Subiao Chen, Ling Lin, Zhengyu Xiao

**Affiliations:** 1 Department of Rheumatology, the first Affiliated Hospital, Shantou University Medical College, Shantou, Guangdong, China; 2 Department of Rheumatology of Shantou University Medical College, Shantou, Guangdong, China; University College London, UNITED KINGDOM

## Abstract

**Objective:**

This study aimed to assess the relationship between bone marrow edema (BME) on magnetic resonance imaging (MRI) and bone mineral density (BMD) in patients with ankylosing spondylitis (AS).

**Methods:**

The study included 333 patients with AS who underwent BMD measurements and axial MRI. Additionally, 106 normal controls were included. The modified New York criteria were used as the classification criteria of AS. Clinical, laboratory, and imaging data were collected and analyzed. Lumbar spine and proximal femur BMD were assessed using dual-energy X-ray absorptiometry. Low BMD was defined by a Z-score ≤-2. The Spondyloarthritis Research Consortium of Canada (SPARCC) MRI index was used to assess inflammation at the sacroiliac joint (SIJ) and spine.

**Results:**

Among the 333 patients, the male:female ratio was 4.6:1, mean patient age was 28.5±10.6 years, and mean disease duration was 7.3±6.8 years. The prevalences of low BMD, osteopenia, and osteoporosis were significantly higher among AS patients than among controls (19.8%, 62.8%, and 5.7% vs. 4.7%, 33.0%, and 0%, respectively, *P* = 0.000). The BMD values were significantly lower and prevalences of low BMD at both the spine and femur were significantly higher among patients with BME on MRI than among those without BME. Multivariate logistic regression analysis showed that male sex (OR 3.87, 95% CI 1.21–7.36, *P* = 0.023), high ASDAS-CRP score (OR 2.83, 95% CI 1.36–4.76, *P* = 0.015), the presence of BME on sacroiliac MRI (OR 2.83, 95% CI 1.77–6.23, *P* = 0.000) and spinal MRI (OR 4.06, 95% CI 1.96–8.46, *P* = 0.000), and high grade of sacroiliitis (OR 2.93, 95% CI 1.82–4.45, *P* = 0.002) were risk factors for low BMD (any site). The SPARCC scores of the SIJ were negatively correlated with femoral BMD (r = -0.22, 95% CI -0.33 to -0.10, *P* = 0.000). Additionally, the SPARCC scores of the spine were negatively correlated with BMD values (r = -0.23, 95% CI -0.36 to -0.09, *P* = 0.003) and Z-scores (r = -0.24, 95% CI -0.36 to -0.12, *P* = 0.001) at the spine.

**Conclusion:**

Low BMD is common in AS patients. BME on MRI is highly associated with low BMD at both the spine and femur.

## Introduction

Ankylosing spondylitis (AS) is a chronic systemic inflammatory disease mainly characterized by inflammation of the axial joints. Osteoporosis (OP) is commonly seen in AS patients, even in the early stage of the disease [1–5], and is associated with an increased risk of fracture. The exact mechanism and causes of bone loss in AS patients have not been fully identified yet. In early AS, inflammation may play a dominant role [1,2,4,6], and in late AS, “bamboo-like” spine and ankylosis of the hip joint result in decreased mobility, which may induce disuse OP [7].

In AS patients, spinal and sacroiliac joint (SIJ) magnetic resonance imaging (MRI) is being used to assess inflammation as an indicator of disease activity [8,9]. Lesions of active inflammation on MRI are depicted as areas of increased signal intensity on T2-weighted images with fat saturation short τ inversion recovery sequences and described as bone marrow edema (BME) [10]. Histopathological studies conducted in both AS and rheumatoid arthritis patients have verified that BME on MRI reflects bone inflammation [11–13]. To date, there has been no large cohort study on the relationship between BME on MRI and bone loss in AS patients.

The aims of this study were to assess the relationship between BME on MRI and bone mineral density (BMD) in AS patients and assess the risk factors associated with low BMD.

## Subjects and methods

### Patients

A total of 333 AS patients from the Department of Rheumatology at the First Affiliated Hospital of Shantou University Medical College were enrolled between February 2005 and June 2014. All the patients underwent BMD measurements and axial MRI (spine/SIJ) at the same time. The modified New York criteria were used as the classification criteria of AS [14]. Fasting and postprandial blood glucose, serum creatinine, and parathyroid hormone levels were assessed in all the patients to exclude common comorbidities (diabetes, chronic kidney disease, and hyperparathyroidism, respectively) that can affect bone metabolism. At baseline, 173 patients had not been treated with any anti-rheumatic drug. The remaining 160 patients had been treated with conventional anti-rheumatic drugs (non-steroidal anti-inflammatory drugs and one to two conventional disease-modifying anti-rheumatic drugs), as well as calcium and vitamin D supplements regularly for more than three months. Three patients had anterior uveitis more than one year before enrollment in this study, and none of the other patients had extra-articular manifestations previously. A total of 106 sex- and age-matched healthy volunteers were enrolled as the control group. This study was approved by the Ethics Committee of Shantou University Medical College, and all the patients signed informed consent forms.

### Data collection

The clinical data of patients, including age, age at disease onset, sex, disease duration, body mass index (BMI), Bath ankylosing spondylitis global assessment (BAS-G) score, Bath ankylosing spondylitis disease activity index (BASDAI) score, Bath ankylosing spondylitis functional index (BASFI) score, and ankylosing spondylitis disease activity score (ASDAS), and the laboratory data, including human leucocyte antigen B27 (HLA-B27) levels, erythrocyte sedimentation rate (ESR), and C-reactive protein (CRP) levels, were obtained from the medical records and were analyzed. Disease duration was defined as the time from the first characteristic symptom to BMD examination. Juvenile onset was defined as disease onset before sixteen years old. According to the reference ranges at our hospital, an elevated ESR was defined as an ESR > 20 mm/h, and an elevated CRP level was defined as a CRP level > 8 mg/L. ASDAS-ESR and ASDAS-CRP were calculated as follows: ASDAS-ESR = 0.079 × total back pain + 0.113 × BAS-G + 0.086 × pain and swelling from peripheral arthritis + 0.069 × duration of morning stiffness + 0.293 × √(ESR); ASDAS-CRP = 0.121 × total back pain + 0.110 × BAS-G + 0.073 × pain and swelling from peripheral arthritis + 0.058 × duration of morning stiffness + 0.579 × ln (CRP + 1) [15].

Axial MRI (spine/SIJ), spinal anteroposterior and lateral radiography, and pelvic anteroposterior radiography were performed to assess inflammatory and structural changes. Hip involvement was assessed according to both clinical manifestations (hip ache) and imaging changes (BME on MRI/structural damage on radiography). Sacroiliitis was graded according to the Modified New York criteria for AS [14]. Spine syndesmophyte formation means a bony growth originating inside the intervertebral ligaments. Bony changes with an angle < 45° to the anterior vertebral side were defined as syndesmophytes, in contrast with changes with an angle of > 45°, which were defined as ambiguous syndesmophytes [16].

MRI of the SIJ was performed using a Power Track 6000 1.5T magnet system (Philips) with an appropriate body coil. Sequences were acquired in an oblique coronal plane, parallel to the long axis of the SIJ, with a slice thickness of 4 mm. The following sequences were used: T1-weighted spin-echo pulse sequence, T2-weighted turbo spin-echo sequence, spectral presaturation with inversion recovery (SPIR) sequence, and fast field-echo sequence. All images were evaluated and scored separately by one radiologist and two rheumatologists (DMW and YG). Any disagreement was worked out through discussions or in consultation of senior instructors. BME on MRI was defined according to the Assessment of SpondyloArthritis International Society (ASAS)/Outcome Measures in Rheumatology (OMERACT) MRI Group criteria [8]. According to these criteria, BME is seen as a hyperintense signal on short tau inversion recovery (STIR) images and usually as a hypointense signal on T1 images. The sacral interforaminal bone marrow signal is the reference for assignment of a normal signal in bone. Bone marrow areas were typically located periarticularly (subchondral bone marrow). If only one signal (BME lesion) for each MRI slice suggests active inflammation, the BME lesion should be present on at least two consecutive slices. If only one MRI slice suggests active inflammation, more than one BME lesion should be present on the single slice [8]. For spine MRI, we defined inflammatory lesions on the evidence of anterior/posterior spondylitis in three or more sites [9]. The Spondyloarthritis Research Consortium of Canada (SPARCC) MRI index was used to assess inflammation at the spine (vertebral corner) and SIJ (subchondral edema) [17, 18].

### Bone mineral density assessment

BMD of the lumbar spine (anterior-posterior at L1–L4) and proximal femur (femoral neck, trochanter, and Ward’s triangle) were measured using dual-energy X-ray absorptiometry (DEXA, DMS Lessos, France). All coefficients of variation of short-term precision at the three sites were less than 1.0%. For patients with syndesmophyte formation in the lumbar spine, we did not include the bilateral calcified paraspinal ligament in the calculative site. The BMD was expressed as a T-score or Z-score. T-score (for postmenopausal women and men over 50 years of age) = (measured value − peak value) / standard deviations of BMD for normal adults. Z-score (for premenopausal women, children, and men under 50 years of age) = (measured value − mean value of the same age group) / standard deviations of BMD for the same age group. The World Health Organization classification system [19] was applied to define normal BMD (T/Z-score > -1 SD), osteopenia (-2.5 < T/Z-score ≤ -1), and OP (T/Z-score ≤ -2.5 SD). According to the International Society of Clinical Densitometry (ISCD) (2007) definition [20], a Z-score of ≤ -2 was termed ‘‘low BMD.”

### Statistical analysis

Statistics Package for Social Sciences 19.0 (SPSS 19.0) was employed for statistical analysis. The normality of the measurement data was first tested; a t test was applied to data that was normally distributed and the rank sum test was used for non-normally distributed data when compare the differences between the groups. The chi square test was used for frequency comparisons. Univariate logistic regression analyses were performed to investigate associations between the presence of low BMD and disease-related factors, and then, multivariate binary logistic regression analyses were performed with backward selection, removing variables that showed an association with the outcome measure with a *P*-value above 0.20. Spearman rank correlation analysis was used to determine the relation between SPARCC scores and Z scores of femoral and spinal BMD. *P* Values < 0.05 were considered statistically significant.

## Results

### Bone mineral density in the AS and normal control groups

Among the 333 patients, 273 were male and 60 were female patients (male:female ratio, 4.6:1). The mean patient age was 28.5±10.6 years, and the mean disease duration was 7.3±6.8 years. Among the 106 normal controls, 87 were male and 19 were female individuals (male:female ratio, 4.6:1). The mean age was 29.7±11.2 years. There was no significant difference in age and percentage of male individuals between the AS patients and normal controls. The prevalences of low BMD, osteopenia, and OP were significantly higher in the AS group than in the control group (19.8%, 62.8%, and 5.7% vs. 4.7%, 33.0%, and 0%, respectively; *P* = 0.000). In the AS group, low BMD was more common at the proximal femur than at the lumbar spine (16.5% vs. 6.3%). The prevalence of low BMD at the femur was higher in patients with hip involvement than in those without hip involvement (23.5% vs. 14.7%). There was no significant difference in the prevalence of low BMD at the spine between patients with and those without syndesmophyte formation (6.2% vs. 6.3%). Although none of the patients had vertebral compression fracture, one patient experienced a fracture at the middle distal femur after falling down a flight of stairs.

### Characteristics of patients with low BMD

Table 1 compares the baseline characteristics of patients with and those without low BMD (Z-score ≤ -2 at any site). The results showed that the ESR, CRP and ASDAS-CRP levels were higher, the frequency of BME on MRI and the SPARCC MRI scores at both the spine and SIJ were also higher among patients with low BMD (n = 66) than among those without low BMD (n = 267) (*P*<0.05).

**Table 1:** Characteristics of the patient groups with normal or low BMD.

	Normal BMD (N = 267)	Low BMD (N = 66)	*P*
Age (years)	28.7±10.9	27.8±9.6	0.724 ^#^
Male gender n (%)	211 (79.0)	62 (93.9)	**0.005**
Disease duration (years)	7.3±7.1	7.3±5.2	0.301 ^#^
BMI (kg/m^2^)	21.6±3.7	19.7±3.2	**0.000** ^#^
Juvenile onset n (%)	80 (30.0)	25 (37.9)	0.215
HLA-B27 (+) n/N (%)	195/227 (85.9)	48/59 (81.4)	0.384
BAS-G	4.1±2.1	4.4±2.4	0.472 *
BASDAI	2.8±2.0	3.0±2.0	0.524 ^#^
BASFI	1.5±1.8	2.1±2.3	**0.047** ^#^
ESR (mm/1h)	19.9±17.6	25.5±18.9	**0.005** ^#^
Elevated ESR n/N (%)	96/266 (36.1)	34/65 (52.3)	**0.016**
CRP (mg/L)	12.1±14.9	19.7±19.8	**0.000** ^#^
Elevated CRP n/N (%)	121/264 (45.8)	43/65 (66.2)	**0.003**
ASDAS-ESR	2.2±0.9	2.5±1.0	0.088 ^#^
ASDAS-CRP	2.4±1.0	2.7±1.0	**0.047** ^*^
BME on sacroiliac MRI n/N (%)	169/250 (67.6)	52/61 (85.2)	**0.006**
SPARCC MRI index of SIJ	13.4±14.6	19.0±14.4	**0.004** ^#^
BME on lumbar spine MRI n/N (%)	55/145 (37.9)	25/38 (65.8)	**0.002**
SPARCC MRI index of spine	5.4±9.7	12.8±12.6	**0.001** ^#^
Sacroiliitis (grade) ≥ 3 n (%)	114 (42.7)	48 (72.7)	**0.000**
Hip involvement n (%)	50 (18.7)	18 (27.3)	0.123
Syndesmophyte formation n (%)	48 (18.0)	17 (25.8)	0.153
Course of treatment (years)	1.6±3.1	1.5±2.3	0.577 ^#^

These data are expressed as mean ± standard deviation. *P* ^*^ data was normally distributed and t test was applied; *P* ^#^ data was non-normally distributed and the rank sum test was applied. BMD bone mineral density; BMI body mass index; HLA human leucocyte antigen; BAS-G Bath ankylosing spondylitis global assessment; BASDAI Bath ankylosing spondylitis disease activity index; BASFI Bath ankylosing spondylitis functional index; ESR erythrocyte sedimentation rate; CRP C-reactive protein; ASDAS ankylosing spondylitis disease activity score; BME bone marrow edema; MRI magnetic resonance imaging; SPARCC Spondyloarthritis Research Consortium of Canada; SIJ sacroiliac joint.

### Presence of BME on SIJ MRI and low BMD

Among the 333 patients, 311 underwent SIJ MRI and BMD examination at the same time. BME was present on MRI in 221 (71.1%) patients. As showed in Table 2, BAS-G, BASFI, ASDAS-ESR, and ASDAS-CRP scores and ESR and CRP levels were higher; BMD values at the femur were lower; and the prevalences of low BMD, osteopenia, and OP at the femur were higher among patients with BME on SIJ MRI than among those without BME on SIJ MRI.

**Table 2:** Comparison of BMD between patients with or without BME on SIJ MRI.

	BME on SIJ MRI (N = 221)	Non BME on SIJ MRI (N = 90)	*P*
Age (years)	26.5±9.6	33.0±12.0	**0.000** ^**#**^
Male gender n (%)	184 (83.3)	72 (80.0)	0.495
Disease duration (years)	6.0±5.5	10.4±8.8	**0.000** ^**#**^
BMI (kg/m^2^)	20.6±3.5	21.7±3.7	**0.000** ^**#**^
BAS-G	4.3±2.2	3.7±1.8	**0.029** ^**#**^
BASDAI	2.9±2.0	2.4±1.6	0.259 ^**#**^
BASFI	1.7±2.1	0.9±1.2	**0.014** ^**#**^
ESR (mm/1h)	21.6±17.8	17.7±17.6	**0.019** ^**#**^
Elevated ESR n/N (%)	94/220 (42.7)	23/89 (25.8)	**0.006**
CRP (mg/L)	14.8±17.7	10.1±12.0	**0.013** ^**#**^
Elevated CRP n (%)	120/218 (55.0)	33/89 (37.1)	**0.004**
ASDAS-ESR	2.3±1.0	2.0±0.8	**0.019** ^**#**^
ASDAS-CRP	2.5±1.0	2.2±0.8	**0.025** ^**#**^
Low BMD in femur n (%)	44 (19.9)	6 (6.7)	**0.004**
Osteopenia in femur n (%)	142 (64.3)	43 (47.8)	**0.000**
OP in femur n (%)	12 (5.4)	3 (3.3)	0.567
BMD of femur (g/cm^2^)	0.749±0.117	0.811±0.121	**0.000** ^*^

These data are expressed as mean ± standard deviation. *P* ^*^ data was normally distributed and t test was applied; *P* ^#^ data was non-normally distributed and the rank sum test was applied. BMD bone mineral density; BME bone marrow edema; SIJ sacroiliac joint; MRI magnetic resonance imaging; BMI body mass index; BAS-G Bath ankylosing spondylitis global assessment; BASDAI Bath ankylosing spondylitis disease activity index; BASFI Bath ankylosing spondylitis functional index; ESR erythrocyte sedimentation rate; CRP C-reactive protein; ASDAS ankylosing spondylitis disease activity score; OP osteoporosis.

### Presence of BME on spinal MRI and low BMD

Among the 333 patients, 183 underwent spinal MRI and BMD examination at the same time. BME was present on MRI in 80 (43.7%) patients. Table 3 compares the characteristics of patients with and those without BME on spinal MRI. The BAS-G, BASDAI, BASFI, ASDAS-ESR, and ASDAS-CRP scores and CRP levels were significantly higher among patients with BME on spinal MRI than among those without BME on spinal MRI. When comparing lumbar BMD and the prevalence of OP between the two groups, we excluded patients with syndesmophyte formation to avoid an artifactual increase in BMD. The results showed that BMD values at the spine were significantly lower and the prevalences of low BMD and osteopenia at the spine were higher among patients with BME on spinal MRI than among those without BME on spinal MRI.

**Table 3:** Comparison of BMD between patients with or without BME on spine MRI.

	BME on spine MRI (n = 80)	Non BME on spine MRI (n = 103)	*P*
Age (years)	30.3±10.0	30.6±11.7	0.886 ^*^
Male gender n (%)	68 (85.0)	84 (81.6)	0.538
Disease duration (years)	8.8±5.9	8.7±8.4	0.181 ^**#**^
BMI (kg/m^2^)	20.8±3.5	21.7±3.9	0.106 ^**#**^
BAS-G	4.5±2.1	3.7±2.1	**0.016** ^*^
BASDAI	3.1±1.9	2.3±1.7	**0.011** ^**#**^
BASFI	1.7±1.8	1.0±1.3	**0.008** ^**#**^
ESR (mm/1h)	21.5±18.0	19.9±16.7	0.531 ^**#**^
Elevated ESR n/N (%)	33/79 (41.8)	40/103 (38.8)	0.689
CRP (mg/L)	15.4±16.7	9.8±13.2	**0.000** ^**#**^
Elevated CRP n/N (%)	48/79 (60.8)	37/103 (35.9)	**0.001**
ASDAS-ESR	2.4±1.0	2.0±0.8	**0.015** ^**#**^
ASDAS-CRP	2.7±1.0	2.1±0.8	**0.000** ^**#**^
Low BMD in spine n/N ^$^ (%)	7/58 (12.1)	1/78 (1.3)	**0.021**
Osteopenia in spine n/N ^$^ (%)	26/58 (44.8)	16/78 (20.5)	**0.002**
OP in spine n/N ^$^ (%)	1/58 (1.7)	0	0.426
BMD of spine ^$^ (g/cm^2^)	0.841±0.124	0.915±0.129	**0.001** ^*^

These data are expressed as mean ± standard deviation. *P* ^*^ data was normally distributed and t test was applied; *P* ^#^ data was non-normally distributed and the rank sum test was applied. BMD bone mineral density; BME bone marrow edema; MRI magnetic resonance imaging; BMI body mass index; BAS-G Bath ankylosing spondylitis global assessment; BASDAI Bath ankylosing spondylitis disease activity index; BASFI Bath ankylosing spondylitis functional index; ESR erythrocyte sedimentation rate; CRP C-reactive protein; ASDAS ankylosing spondylitis disease activity score; OP osteoporosis.

^$^ Only calculated the patients without syndesmophytes formation at the lumbar spine.

### Variables associated with low BMD

Multivariate logistic regression with stepwise selection of variables statistically correlated in univariate analysis (Table 4) showed that for low BMD (any site), male sex (OR 3.87, 95% CI 1.21–7.36, *P* = 0.023), high ASDAS-CRP score (OR 2.83, 95% CI 1.36–4.76, *P* = 0.015), the presence of BME on sacroiliac MRI (OR 2.83, 95% CI 1.77–6.23, *P* = 0.000) and spinal MRI (OR 4.06, 95% CI 1.96–8.46, *P* = 0.000), and high grade of sacroiliitis (OR 2.93, 95% CI 1.82–4.45, *P* = 0.002) were risk factors, while high BMI (OR 0.91, 95% CI 0.71–0.97, *P* = 0.011) was a protective factor.

**Table 4:** Variables associated with low BMD in univariate analysis.

	Low BMD in total		Low BMD in femur		Low BMD in spine	
	OR	95% CI	OR	95% CI	OR	95% CI
Male gender	4.11^#^	1.44–11.79	4.36*	1.68–9.23	2.17	0.49–9.57
Age	0.99	0.97–1.02	0.99	0.96–1.02	0.96	0.91–1.01
Disease duration	1.00	0.96–1.04	1.00	0.96–1.05	0.98	0.91–1.05
BMI	0.85^#^	0.78–0.93	0.83^#^	0.75–0.92	0.86*	0.74–0.99
Juvenile onset	1.43	0.81–2.50	1.43	0.78–2.60	4.86^#^	1.90–12.43
HLA-B27	0.72	0.34–1.52	0.86	0.37–2.00	0.69	0.22–2.16
BASDAI	1.05	0.90–1.23	1.08	0.92–1.27	1.19	0.92–1.55
BASFI	1.16*	1.01–1.35	1.17*	1.00–1.36	1.24	0.99–1.55
Elevated ESR	1.94*	1.12–3.36	2.21^#^	1.23–3.99	3.35*	1.31–8.53
Elevated CRP	2.31^#^	1.31–4.08	1.90*	1.04–3.46	6.66^#^	1.92–13.07
ASDAS-ESR	1.31	0.96–1.78	1.37	0.99–1.90	1.92*	1.16–3.18
ASDAS-CRP	1.39*	1.03–1.90	1.45*	1.05–2.00	2.20^#^	1.32–3.66
Sacroiliac BME on MRI	2.77^#^	1.30–5.90	3.48^#^	1.43–8.49	2.26	0.64–7.97
Spine BME on MRI	3.15^#^	1.49–6.66	3.06^#^	1.41–6.63	4.57^#^	1.82–16.41
Sacroiliitis (grade) ≥ 3	3.58^#^	1.98–6.48	3.41^#^	1.80–6.46	2.22	0.87–5.64
Hip involvement	1.63	0.87–3.03	2.78*	1.53–3.43	1.61	0.60–4.33
Calcium and vitamin D intake	1.49	0.86–2.57	0.60	0.24–1.50	1.06	0.59–1.89

* *P* < 0.05

# *P* < 0.01

BMD bone mineral density; AS ankylosing spondylitis; OR odd ratio, CI confidence interval; BMI body mass index; HLA human leucocyte antigen; BASDAI Bath ankylosing spondylitis disease activity index; BASFI Bath ankylosing spondylitis functional index; ESR erythrocyte sedimentation rate; CRP C-reactive protein; ASDAS ankylosing spondylitis disease activity score; BME bone marrow edema; MRI magnetic resonance imaging; SPARCC Spondyloarthritis Research Consortium of Canada; SIJ sacroiliac joint.

For low femoral BMD, male sex (OR 4.36, 95% CI 1.68–9.23, *P* = 0.014), the presence of BME on sacroiliac MRI (OR 3.43, 95% CI 2.17–8.46, *P* = 0.000) were the main risk factors. For low spinal BMD, the presence of BME on spinal MRI (OR 4.06, 95% CI 1.94–9.02, *P* = 0.000) was the main risk factor.

Correlation analysis showed that the SPARCC scores of the SIJ were negatively correlated with femoral BMD (r = -0.22, 95% CI -0.33 to -0.10, *P* = 0.000). Additionally, the SPARCC scores of the spine were negatively correlated with BMD (r = -0.23, 95% CI -0.36 to -0.09, *P* = 0.003) and Z-scores (r = -0.24, 95% CI -0.36 to -0.12, *P* = 0.001) at the spine.

## Discussion

OP is a common complication of AS, with a reported prevalence of 9–36% [1, 5, 21–25]. The prevalences of OP (based on DEXA measurements) in patients within the first decade after diagnosis are 16% and 13% at the lumbar spine and femoral neck, respectively [26]. In this study, 19.8% of the AS patients had low BMD, and the prevalence of OP was 5.7%. The lower prevalence of OP in our study might be due to the younger age and shorter disease duration in our study than in the previous studies.

The prevalence of low BMD was significantly higher in AS patients than in healthy controls, indicating that bone loss is common in AS patients. The most frequently affected site with low BMD was the proximal femur. Logistic regression analysis showed that hip involvement was a risk factor for low BMD at the femur. The association between hip involvement and bone loss might be explained by inflammation at the hip. However, both pain at the early stage and hip ankylosis at the late stage will decrease the mobility of patients, which may induce disuse OP [7].

In this study, 19.5% (65/333) of the AS patients had syndesmophyte formation at the lumbar spine. The low prevalence of OP at the lumbar spine in our study might be explained by an artifactual increase in BMD resulting from the syndesmophyte formation. Previous studies in long-standing AS patients also showed that reduced BMD is reflected by low hip BMD, and high lumbar spine BMD is related to an artifactual increase related to either the presence of syndesmophytes or periosteal bone formation [7, 27]. Some investigators suggested lateral lumbar spine BMD measurements in patients with syndesmophytes [28]; however, the precision of DEXA measurements is reasonably lower at the lateral spine than at the AP spine or proximal femur [22, 29]. Therefore, the proximal femur is the preferred site of BMD determination in late AS using DEXA.

Our study confirmed the influence of inflammation, especially local bone inflammation, on BMD. Disease activity was higher and the presence of BME at the lumbar spine and SIJ on MRI was more common among patients with low BMD than among those without low BMD. The mean BMD values at both sites were significantly lower among patients with BME on MRI. Logistic regression analysis confirmed that the presence of BME on MRI was highly associated with low BMD at both the SIJ and spine. Correlation analysis also showed that SPARCC MRI scores were negatively correlated with BMD. All these results support the view that inflammation is an etiological factor of bone loss in AS patients [1, 2, 4, 6, 30–32]. Recently, a large cohort study of patients with early inflammatory back pain showed that BME on MRI was highly associated with low BMD at both the spine and SIJ [4], but only 28.9% of the study population had radiographic sacroiliitis. Gürkan Akgöl et al reported that non-radiographic axial SpA (nr-axSpA) patients (n = 46) had significant bone loss in the lumbar spine when compared with that in patients with mechanical low back pain and mentioned that low bone mass is associated with ongoing spinal inflammation on MRI in nr-axSpA patients [33]. Another 1-year longitudinal study in patients with early IBP showed that bone loss in the hip was associated with high baseline CRP levels and baseline presence of BME on SIJ MRI [2]. Therefore, aggressive intervention at the progression stage in AS (especially those with BME on MRI) may be effective for preventing bone loss through not only increased mobility related to pain relief but also a direct anti-inflammation effect on bone.

In conclusion, this study provides evidence that low BMD is common in AS patients. Additionally, local bone inflammation (BME on MRI) at both the SIJ and spine is the main risk factor for bone loss.

## Supporting information

S1 File Low BMD in ankylosing spondylitis. (XLS)

S2 File PLOS ONE clinical studies checklist. (DOCX)
